# Anaemia and malaria

**DOI:** 10.1186/s12936-018-2509-9

**Published:** 2018-10-19

**Authors:** Nicholas J. White

**Affiliations:** 1 Faculty of Tropical Medicine, Mahidol University, Bangkok, Thailand; 2 Centre for Tropical Medicine and Global Health, Nuffield Department of Medicine, University of Oxford, Oxford, UK

## Abstract

Malaria is a major cause of anaemia in tropical areas. Malaria infection causes haemolysis of infected and uninfected erythrocytes and bone marrow dyserythropoiesis which compromises rapid recovery from anaemia. In areas of high malaria transmission malaria nearly all infants and young children, and many older children and adults have a reduced haemoglobin concentration as a result. In these areas severe life-threatening malarial anaemia requiring blood transfusion in young children is a major cause of hospital admission, particularly during the rainy season months when malaria transmission is highest. In severe malaria, the mortality rises steeply below an admission haemoglobin of 3 g/dL, but it also increases with higher haemoglobin concentrations approaching the normal range. In the management of severe malaria transfusion thresholds remain uncertain. Prevention of malaria by vector control, deployment of insecticide-treated bed nets, prompt and accurate diagnosis of illness and appropriate use of effective anti-malarial drugs substantially reduces the burden of anaemia in tropical countries.

## Background

Malaria is the most important parasitic disease of man [1]. It is a major cause of anaemia in endemic areas, and in areas of higher transmission malaria is one of the most common reasons for blood transfusion. Six species of the genus *Plasmodium* infect humans commonly, and all cause anaemia. Most malaria attributable deaths and severe disease are caused by *Plasmodium falciparum.* The majority of fatalities occur in the community. The World Health Organization (WHO) has estimated that there were some 216 million cases and 445,000 deaths from malaria in 2016 [2]. A significant proportion of these deaths resulted directly or indirectly from anaemia.

## Epidemiology of malaria and anaemia

**Fig. 1:**
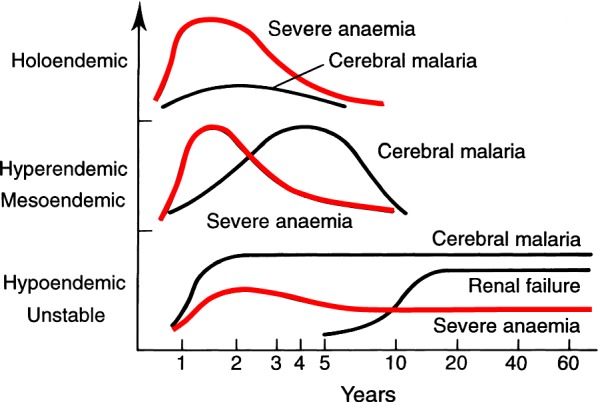
Relationship of severe falciparum malaria manifestations to age at different levels of malaria transmission

**Fig. 2:**
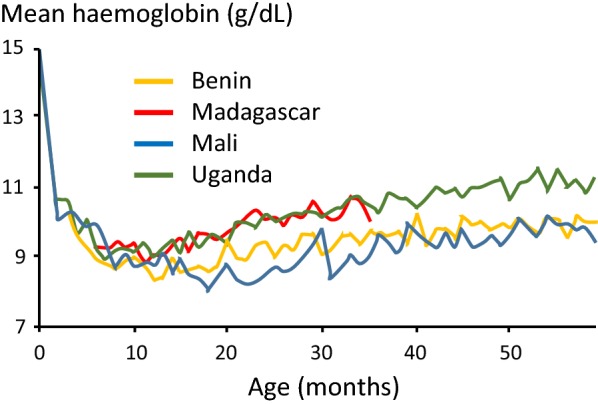
Age patterns of average haemoglobin concentrations in young children in Demographic and Health Surveys (DHS) from four African countries with moderate to high malaria transmission (Reproduced from Crawley J, with permission [11])

The clinical consequences of malaria, and in particular the prevalence of anaemia, depend on the intensity of malaria transmission (Fig. 1). The main determinants of malaria transmission intensity are the density, longevity, biting habits, and efficiency of the local mosquito vectors [1]. In high transmission settings people may receive as much as one infectious bite each day, so the entire population is infected repeatedly, but it is young children who bear the brunt of the disease, and most are anaemic [3–12]. As the child grows a disease controlling immunity develops such that by adolescence and adulthood nearly all malaria infections are asymptomatic. The prevalence of anaemia declines (Fig. 2). Thus, apparently healthy individuals carry malaria parasites in their blood. These infections can persist for many months. This persistent asymptomatic parasite carriage reduces the operational diagnostic specificity of a positive microscopy or rapid test result as febrile illness in a parasitaemic individual may be caused by other infections. Meta-analyses of the relationship between malaria and anaemia are confounded by the non-specificity of parasitological diagnosis in high transmission settings, widespread self-treatment of febrile illness, inability to characterize preceding infections and thus recurrences in point prevalence surveys, and frequent concomitant haemoglobinopathies, nutritional deficiencies (particularly iron deficiency), and intestinal helminth infections—all of which contribute variably to anaemia [10, 11]. Thus, in higher transmission settings malaria increases the risk of anaemia in the entire population with the greatest impact in young children, and in particular infants [3]. In lower transmission settings symptomatic malaria and resulting anaemia may occur at all ages, although it is children and pregnant women who are more likely to be anaemic. At all levels of transmission malaria (all species) is an important contributor to maternal anaemia during pregnancy, and poor birth outcomes [13]. Falciparum malaria is a direct cause of maternal mortality in lower transmission settings and an indirect cause by contributing to anemia in higher transmissions settings [14]. Anti-malarial drug resistance causing recrudescent infections increases the prevalence and the severity of malaria anaemia.

### Clinical patterns

Constant, frequent, year-round malaria reflects *stable transmission*. In the sub-Sahel region across Africa from Senegal to Sudan there is intense malaria transmission, but this is largely confined to the 3–4 month rainy season. During this period young children are commonly anaemic and frequently present to hospitals and health centres with severe anaemia. In contrast in areas where malaria transmission is low, erratic, or focal (often termed *unstable transmission*), protective immunity from malaria is not acquired, and symptomatic malaria may occur at all ages. In such areas changes in environmental, economic, or social conditions, such as heavy rains following drought or large population movements together with a breakdown in malaria control and prevention services (often resulting from conflict) can result in epidemics of malaria with considerable mortality among all age groups [1]. Recent improvements in malaria control have reduced malaria transmission in many areas and increased heterogeneity in malaria epidemiology. Unfortunately, there is evidence that this recent progress has stalled, and malaria incidence in some parts of the tropics has started to rise again.

**Fig. 3:**
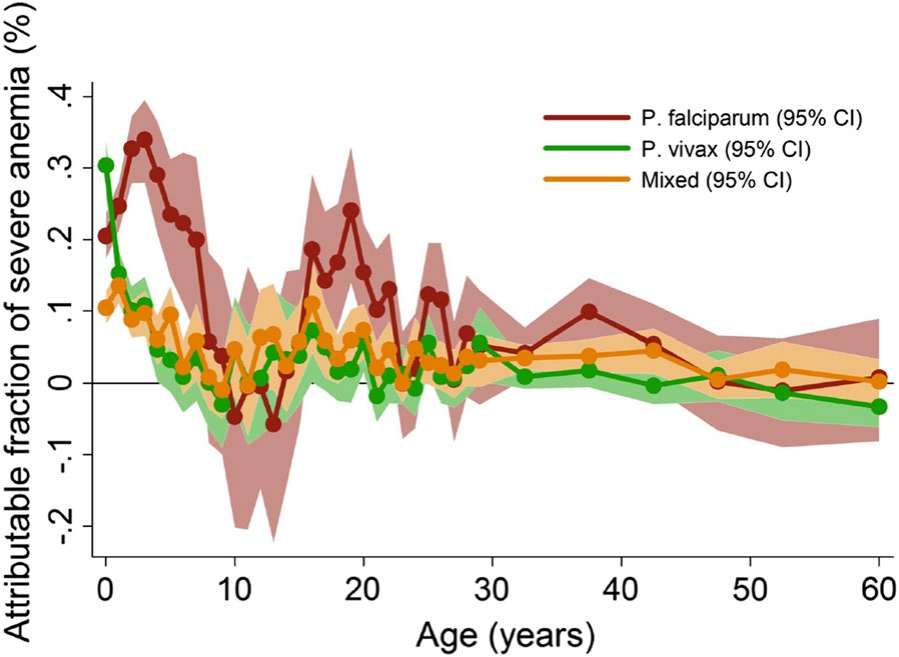
Estimated contributions of falciparum and vivax malaria to severe anaemia in Papua. Shows adjusted population attributable fractions with 95% confidence bands of severe anaemia (Hb < 5 g/dL) by Plasmodium species and by age (From Douglas et al. with permission [9])

In areas of moderate or high transmission malaria infections (both *P. falciparum* and *Plasmodium vivax*) in pregnancy cause maternal anaemia, intrauterine growth retardation and prematurity [1]. The newborn starts at a developmental disadvantage with a lowered haemoglobin [15]. The physiological decline in haemoglobin in early infancy is exaggerated and plateaus around 9 g/dL on average around 9 months. Thereafter, there is a slow but steady rise in haemoglobin concentrations punctuated by acute reductions associated with symptomatic malaria infections [11] (Figs. 2 and 3).

### Concomitant contributors to anaemia

Poor malaria control is often associated with weak health structures, and high prevalences of other infectious diseases and nutritional deficiencies, all of which contribute to anaemia. In Malawi, where transmission of malaria is moderate to high and childhood malaria is very common, bacterial infections, HIV infection, hookworm and deficiencies in vitamins A and B12 were all independently associated with severe anaemia [10]. Malaria has also selected for haemoglobinopathies and other inherited red cell abnormalities [notably glucose 6 phosphate dehydrogenase (G6PD) deficiency] which provide some protection against the pathological consequences of malaria but themselves contribute to anaemia [12, 16]. Sickle cell anaemia is common in most of sub-Saharan Africa (birth prevalence 1–2%) and is a major cause of severe anaemia, commonly provoked by malaria illness [17]. Patients with G6PD deficiency are also at increased risk of severe malarial anaemia [18]. Dissecting and quantitating the individual contributions of these various genetic factors to malaria anaemia overall is difficult.

### Relationship of anaemia to transmission intensity

In most of Asia and the Americas malaria transmission is low and seasonal. In Asia, the prevalences of *P. falciparum* and *P. vivax* malaria overall are approximately equal [2]. In the Americas *P. vivax* predominates. In these areas people commonly receive ≤ 1 infectious bite per year (the entomological inoculation rate; EIR). Malaria is usually associated with mild anaemia, although where resources are limited, then even at these low transmission intensities *P. vivax* may still cause severe anaemia in children because each sporozoite inoculation can result in multiple relapses. Transmission intensities are much higher in many parts of sub-Saharan Africa, where *P. falciparum* predominates, and in lowland New Guinea where both *P. falciparum* and *P. vivax* are prevalent (Fig. 3); EIRs may reach as high as 1000/year in some areas of Africa. In such high transmission settings where everyone is infected, morbidity and mortality from malaria are considerable. Newborns have low birthweight and infant mortality is high [1–6]. Babies and young children suffer repeated symptomatic infections with high rates of asymptomatic parasitaemia between these episodes. They are often chronically anaemic with palpably enlarged spleens. There is an increased mortality both from malaria itself, and also indirectly from other infections which repeated malaria predisposes to. If the child survives then by adulthood most malaria infections are asymptomatic.

### Contribution of malaria to anaemia

The best estimates of the causal contribution of malaria to anaemia in a particular setting come from randomized trials of malaria control interventions [19, 20]. A review of 29 community-based studies of insecticide-treated nets (ITNs), anti-malarial chemoprophylaxis, and insecticide residual spraying found that among children < 5 years exposed to between 1 and 2 years of malaria control, mean relative risk for a haemoglobin concentration < 11 g/dL was 0.73 (95% CI 0.64–0.81), and for a haemoglobin < 8 g/dL was 0.40 (95% CI 0.25–0.55) compared with the control groups not exposed to these malaria interventions [20, 21]. The WHO and the Roll Back Malaria (RBM) Partnership have recommended that anaemia be used as an additional indicator to monitor malaria burden at the community level as malaria control interventions are scaled up nationally. This recommendation is based on results of an extensive review conducted by Korenromp et al. [20] showing that, in areas of stable malaria transmission, the prevalence of moderate-to-severe anaemia (haemoglobin < 8 g/dL) is a more sensitive measure of a reduction in malaria exposure than parasite prevalence, and that it may respond more quickly than mortality as coverage of malaria interventions, such as insecticide-treated bed nets (ITNs), malaria chemoprevention and indoor residual spraying are scaled up [20–22]. In randomized controlled trials, the impact of ITNs on anaemia was more pronounced than on the prevalence of malaria parasitaemia or on the incidence of clinical malaria.

Improved control between 2000 and 2015 has resulted in a reduction in malaria in high transmission areas, a substantial reduction in global malaria attributable mortality, and elimination of malaria from several countries [2, 23, 24].

## Pathogenesis

The pathogenesis of malarial anaemia is multifactorial [1, 25–34]. Malaria is an intraerythrocytic parasite so there is obligatory destruction of red cells containing parasites at schizont rupture. However, a more important contributor is the accelerated destruction of non-parasitized red cells that parallels disease severity [30]. It has been estimated that loss of unparasitized erythrocytes accounts for approximately 90% of the acute anaemia resulting from a single infection. Parasitaemias in falciparum malaria commonly exceed 1% (of red cells parasitized), and in severe disease may exceed 10%. *Plasmodium knowlesi* may also cause hyperparasitaemia, but parasite densities in the other human malarias very rarely exceed 2% [1]. In severe falciparum malaria there is a heavy parasite burden and anaemia develops rapidly. The main contributor to this usually rapid decline in haematocrit is the haemolysis of unparasitized red cells [30, 35–37]. The ratio of unparasitized red cells to parasitized red cells lost in acute malaria is even higher in *P. vivax* than in *P. falciparum* infections [36].

**Fig. 4:**
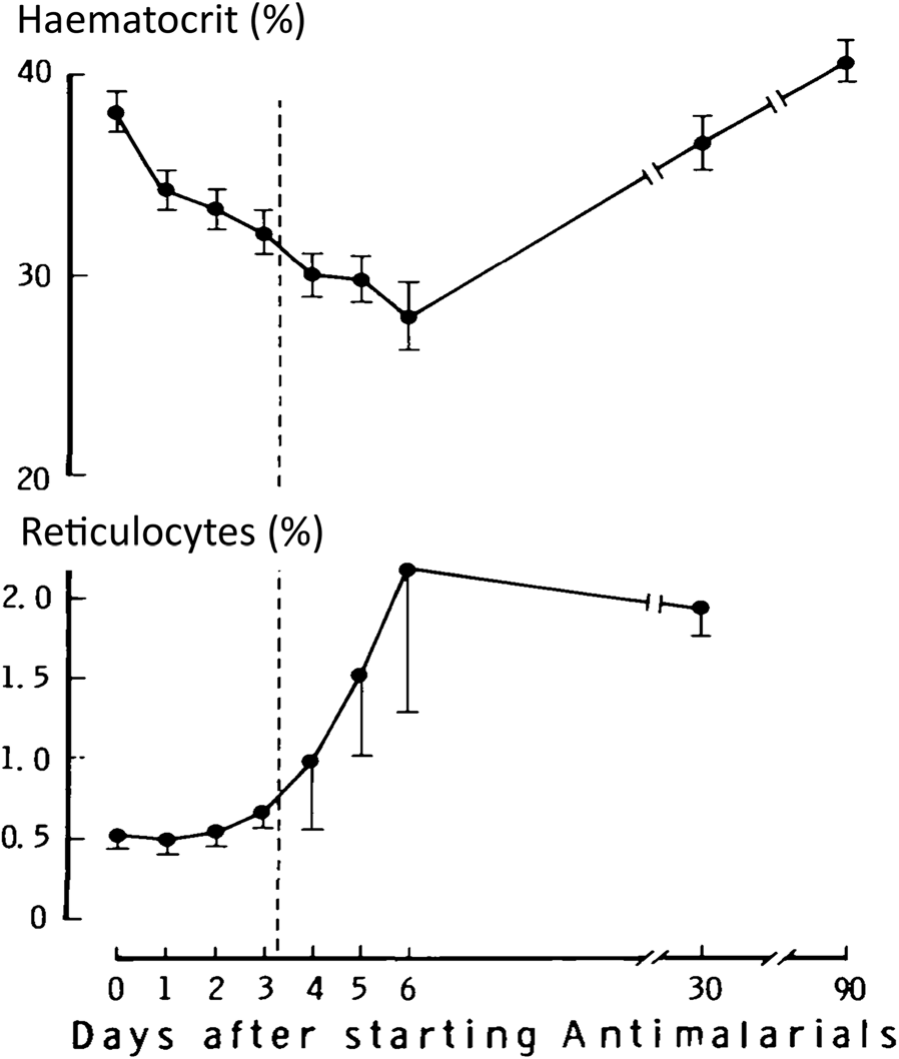
The delayed reticulocyte response to anaemia in Thai adults with acute uncomplicated falciparum malaria [28]. Shows mean and SE values, and dashed line represents time to parasite clearance assessed by microscopy

The haemolytic anaemia of malaria is compounded by bone marrow dyserythropoiesis during and immediately after the acute illness [28, 29, 38, 39]. Bone marrow dyserythropoiesis persists for days or weeks following the start of malaria treatment in acute malaria. As a consequence reticulocyte counts are usually low in the acute symptomatic phase of the disease (Fig. 4). This accounts for the delayed erythropoietic response in acute malaria in low transmission settings. In such settings, the nadir of haemoglobin concentration in acute falciparum malaria is usually around 1 week after presentation with fever [33]. In acute vivax malaria the nadir in haemoglobin is earlier (usually after a few days) [34]. In contrast in higher transmission settings, in the context of some premunition from repeated previous infections, haemoglobin concentrations usually begin to rise immediately following the start of effective anti-malarial treatment. In acute uncomplicated malaria, the resultant anaemia is worse in younger children, and those with protracted infections.

### Bone marrow dysfunction in malaria

Dyserythropoiesis in malaria is thought to be related to intramedullary production of mediators which suppress erythropoiesis (proinflammatory cytokines, nitric oxide, lipoperoxides, bioactive aldehydes) and, in some studies, these have been incriminated in causing red cell precursor apoptosis [38–44]. It has long been observed that dyserythropoiesis and anaemia are associated with intramedullary deposition of malaria pigment (haemozoin). This is the obligatory by-product of intraerythrocytic malaria parasite haemoglobin digestion (and thus haem detoxification). The haemozoin is expelled in the residual body at schizont rupture, and is commonly seen in peripheral blood or bone marrow smears having been phagocytosed by neutrophils and monocytes/macrophages. Indeed high proportions of peripheral blood monocytes containing malaria pigment reflect higher parasite burdens and are associated with anaemia in African children [38, 39]. Native haemozoin comprises a scaffold of crystalline cyclic haem dimers (α-hematin), but it also contains large amounts of associated polyunsaturated fatty acids (PUFA). These PUFA are non-enzymatically peroxidized and broken down by the haemozoin associated iron to bioactive terminal hydroxyaldehydes such as 4-hydroxy 2-nonenal (HNE) [42, 43]. Haemozoin has been shown both to induce and to suppress pro-inflammatory cytokine production in different experimental circumstances. The role of the macrophage in the pathological process has been controversial—while several studies have suggested that macrophages contribute to the inhibition of erythropoiesis either indirectly or directly by generating oxidative stress—others have suggested they exert an overall protective effect against a direct toxic effect of native haemozoin in inducing premature apoptosis of red cell progenitor cells [38, 40, 44]. In a different experimental system inhibition of erythropoiesis rather than apoptosis has been observed. The bioactive aldehyde HNE, generated by the haem iron mediated peroxidation of PUFA, was shown to be an important mediator of this effect [42, 43].

Severe malarial anaemia in African children has been associated with the 238A TNF promoter genetic polymorphism and low levels of the anti-inflammatory cytokine IL-10 [45, 46]. Malaria anaemia in African children is also associated with the haptoglobin 2-2 genotype, an association postulated to reflect the reduced ability of the Hp2-2 polymer to scavenge free haemoglobin-iron following malaria-induced haemolysis [47]. Serum erythropoetin levels are usually elevated in malarial anaemia, although in some series it has been suggested that the degree of elevation was insufficient for the reduction in haemoglobin [31, 32, 48].

### Reduced red cell deformability

**Fig. 5:**
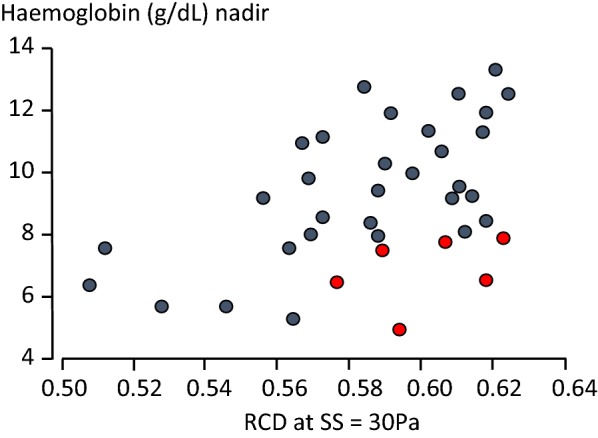
Correlation between admission values of mean red blood cell deformability (RCD) at a shear stress (SS) of 30 Pa and the lowest haemoglobin (Hb) concentration reached during hospitalization in Thai patients with severe falciparum malaria (correlation coefficient 0.49, *P *= 0.002). When patients with a microcytic anaemia (MCV,80fL: red circles) were excluded, the correlation coefficient was 0.64, *P *< 0.001) [49]

In severe malaria caused by *P. falciparum* or by *P. knowlesi* the entire red cell population becomes less deformable [49–52]. When red cell deformability was assessed at the shear stresses encountered in the splenic sinusoids (30 Pa) reduced deformability was correlated significantly with the reduction in haemoglobin [49] (Fig. 5). The mechanisms responsible for reduced uninfected erythrocyte deformability have not been identified with certainty, although there is evidence in acute malaria for increased oxidative damage which might compromise red cell membrane function and reduce deformability [50, 53, 54]. In simian malarias, inversion of the erythrocyte membrane lipid bilayer in uninfected erythrocytes has been reported, but this has not been studied in man [55].

### Antibody and complement binding

**Fig. 6:**
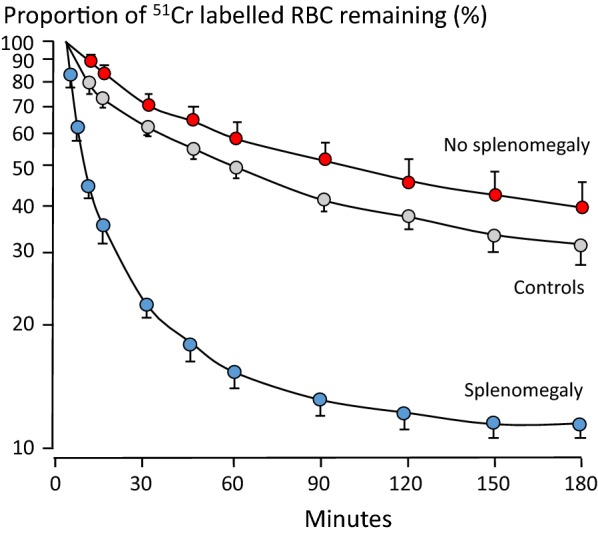
Augmented splenic clearance function for rigid erythrocytes associated with splenic enlargement in Thai adults with acute malaria [61]. Clearance curves of ^51^Cr-labelled heated autologous erythrocytes after intravenous injection to 10 uninfected volunteers, 9 patients with no detectable splenomegaly and 16 with palpable spleens (mean ± SD)

The role of red cell membrane bound antibody (i.e. Coombs’-positive haemolysis) in malarial anaemia is unresolved. Some studies have shown increased red cell immunoglobulin binding in malaria, whereas others have not [56–58]. In the presence of the malaria associated lowered clearance threshold for splenic red cell removal (Fig. 6), increased antibody or complement binding might be difficult to detect. Nevertheless, studies in Kenyan children with severe anaemia have shown increased surface IgG and immune complexes and also deficiencies in the complement regulatory proteins CR1 and CD55 [59, 60]. The circulating erythrocytes from these children were more susceptible to phagocytosis than were those of controls [59].

### The role of the spleen

**Fig. 7:**
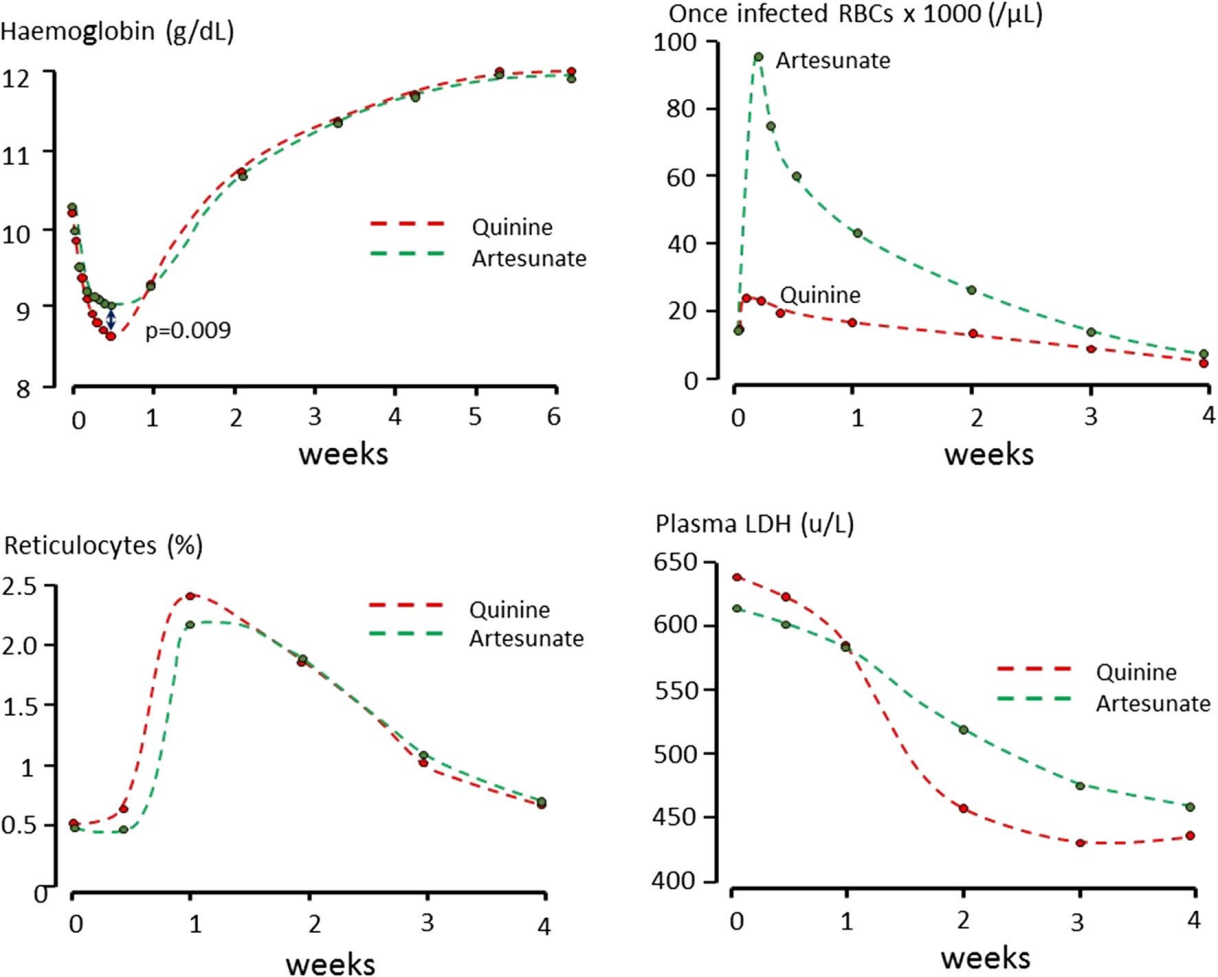
Reduction in haemoglobin concentrations, corresponding increases in pitted erythrocytes, reticulocyte responses and plasma LDH in relation to anti-malarial drug treatment (artesunate or quinine) in African children in Kinshasa, DRC, admitted to hospital with hyperparasitaemic falciparum malaria [70]

In acute malaria the spleen reorganizes and enlarges rapidly. This results in increased clearance capacity and a lowered splenic threshold for the clearance of abnormal erythrocytes, whether because of antibody coating or reduced deformability [52, 61–63]. Thus, the spleen removes large numbers of relatively rigid red cells, which in a healthy uninfected subject would be allowed to remain in the circulation. This results in shortened erythrocyte survival. The reduction in red cell survival is proportional to the severity of the infection. Red cell clearance is unaffected by corticosteroids [64]. The spleen also fulfils its normative function of removing intraerythrocytic particles. In this case, the spleen removes damaged intraerythrocytic parasites (particularly following treatment with an artemisinin derivative) by a process called “pitting” [65]. It then returns the “once parasitized” red cells back to the circulation—but these pitted erythrocytes then have reduced survival [66, 67] (Fig. 7). In non-immune hyperparasitaemic patients the markedly shortened survival of these damaged, once parasitized, erythrocytes may cause a delayed sudden haemolysis, typically 1 to 2 weeks after starting treatment with an artemisinin derivative [67–69]. As it is the killing of these younger circulating parasites which explains much of therapeutic superiority of artesunate over quinine in severe malaria, this deferred haemolysis of once infected erythrocytes may be regarded as the “price” of the life-saving benefit. In higher transmission malaria endemic areas, where most malaria anaemia occurs, clinically significant post-artesunate delayed haemolysis is rare [70] (Fig. 7).

Repeated malaria infections result in splenomegaly and, in some cases, hypersplenism. At its most extreme is a condition called “hyperreactive malarial splenomegaly”, known in the past as “tropical splenomegaly”, in which there is massive splenomegaly, hypersplenism and dilutional anaemia. Some cases progress to B cell malignancy. Untreated the mortality is high, but when caused by malaria, the splenomegaly resolves over weeks or months with effective malaria chemoprophylaxis [71–73].

### Iron deficiency

The interaction between iron and malaria is complex and controversial. Iron deficiency is very common in malaria endemic areas. It causes anaemia and in young children iron deficiency is associated with neurodevelopmental delay. Malaria does not cause iron deficiency, but iron deficiency does reduce the incidence of severe malaria [74]. Nevertheless, iron deficiency and malaria still often coincide in the same patient. Assessment of iron deficiency in acute malaria is confounded by the associated inflammatory response. In some areas, but not others, routine elemental iron supplementation following malaria has been shown to promote recovery from anaemia [75, 76]. Secondary folate deficiency is less common. Neither iron nor folate supplementation reduce childhood mortality in areas of high malaria transmission. Much of the controversy has centred on whether iron (and folate) supplementation actually worsen malaria and increase malaria associated mortality. Some large prospective studies, notably a study conducted on Pemba island which was stopped prematurely [77], have shown increased falciparum malaria morbidity and mortality in elemental iron supplemented children [77–80]. So is it good or bad to provide elemental iron supplementation to children in malaria endemic areas? The risk–benefit assessment, and thus the answer to this question, varies and so is likely to be context specific [81]. Currently, the WHO recommends that daily iron supplementation should be given to infants and young children aged 6–23 months, living in settings where the prevalence of anaemia is 40% or higher in that age group [82], a recommendation that may still leave the younger infants vulnerable [83]. This is not widely implemented. Provision of lower quantities of iron within a food matrix, i.e., fortified food, has been proposed as a safer strategy than non-physiological elemental iron supplementation [84]. In acute malaria hepatocyte production of the key iron regulator hepcidin is increased. This reduces iron uptake, and lowers serum iron [85]. Concentrations of serum ferritin, an acute phase reactant, are also raised. The redistribution of iron in malaria is considered a risk factor for supervening bacterial infections which are associated with malaria in endemic areas [86], and particularly with severe malarial anaemia.

## Diagnosis of malaria

In the clinical assessment of anaemia a diagnosis of acute malaria requires either demonstration of malaria parasites in a thick or thin blood film, or a positive rapid test (RDT). Microscopy or RDTs have a detection threshold of approximately 50 parasites/μL, which also corresponds approximately with the pyrogenic density in non-immune subjects [1]. The RDTs for falciparum malaria usually identify histidine-rich protein 2 (*Pf*HRP2) as the target antigen. *Pf*HRP2 persists in pitted erythrocytes [87] and so these RDTs commonly remain positive for days or weeks after parasite clearance, whereas the *p*LDH-based tests become negative as parasitaemia clears. The RDTs for *P. falciparum* are slightly more sensitive than those for *P. vivax* malaria. Using sufficient volume blood samples PCR methods can now detect parasite densities 1000 times lower than microscopy or RDTs, but because of the high background rates of asymptomatic parasitaemia, even in low transmission settings [88], they are too sensitive for the diagnosis of acute illness (i.e. their predictive value for identifying malaria as the cause of illness is poor). Serology maybe useful in assessing previous malaria exposure, but not in identifying the cause of an individual’s illness [1, 89]. However, in many cases in which malaria causes anaemia the acute infection has resolved or been treated. The epidemiological context is critical to the assessment. In some cases, finding residual malaria pigment in peripheral blood monocytes provides a useful clue to recent infection.

## Definitions of anaemia

As the epidemiology of malaria coincides with the epidemiology of inherited red cell abnormalities, nutritional deficiencies and helminth infections, anaemia is often multifactorial, and the distribution of haemoglobin concentrations in healthy people is lower and broader than in temperate countries. Although a wide, and frankly confusing, variety of definitions of anaemia in general have been proposed, the most commonly used definitions in malaria studies, based on haemoglobin concentrations, are as follows.

Mild anaemia ≤ 11 g/dL

Moderate anaemia ≤ 8 g/dL

Severe anaemia ≤ 5 g/dL

## Measurement

Pallor is readily recognizable clinically, and village health workers can be trained to recognize it, but anaemia is best quantitated by measurement in a capillary or venous blood sample of either the haemoglobin concentration (most widely assessed using the HemoCue^®^ system-a portable spectrophotometric analyser) or the haematocrit using a microcentrifuge. Well-functioning Coulter counters and other types of cell sorters are rarely found in rural areas of the tropics. The relationship between red cell count and haemoglobin or haematocrit is determined by red cell volume. In many areas microcytosis (either from iron deficiency or thalassaemia) is common. Malaria itself does not affect the relationship. The usual conversion factor of 3 for haemoglobin to haematocrit slightly overestimates the haematocrit [90, 91].

In 1810 patients with acute malaria who provided 3254 simultaneous measurements from various time points (ranging from day 0 to day 63), a good fit was obtained using Haematocrit = 5.62 + 2.60 * Haemoglobin [90].

In areas of high malaria transmission where malaria is a major contributor to anaemia in the first years of life the ratio of haemoglobin to haematocrit changes with age [91]. Clinical trials of therapeutic interventions in malaria usually report changes in haemoglobin or haematocrit, whereas large assessments of preventive interventions more commonly report the prevalence of anaemia from cross sectional surveys, or sometimes the incidence of anaemia in cohort studies.

The term moderate anaemia has been used variably in epidemiological studies. For example, in a recent assessment of seasonal chemoprevention in malaria prevention moderate anaemia was defined as < 11 g/dL (and in this study severe anaemia was defined as < 6 g/dL) [92]. In most studies the term anaemia (without specifying severity) refers to < 11 g/dL (although some have also used the < 8 g/dL threshold). The majority of studies have used 5 g/dL to define severe malarial anaemia.

## Clinical features

### Uncomplicated malaria

Malaria is an acute febrile illness. There are no specific clinical features in uncomplicated infections. Although, in general, higher parasitaemias are associated with more severe clinical disease, the relationship is very variable [1]. In falciparum malaria there is sequestration of erythrocytes containing mature parasites in the microcirculation. This causes microvascular obstruction and accounts for much of the pathology of severe disease [93, 94]. Thus, the parasites causing pathology in severe infections are not represented directly by those counted in the peripheral blood smear. Patients can have the majority of their parasites circulating, or sequestered. In the latter case, the peripheral parasitaemia can be low (depending on stage of development and synchronicity). However, the peripheral blood film does provide an indication. In patients with a predominance of circulating parasites, most of the parasites seen in the blood smear are young ring stages, whereas those with a predominantly sequestered biomass usually have more mature trophozoites, many of which contain visible malaria pigment [95]. Significant sequestration does not occur in the other human malarias.

**Fig. 8:**
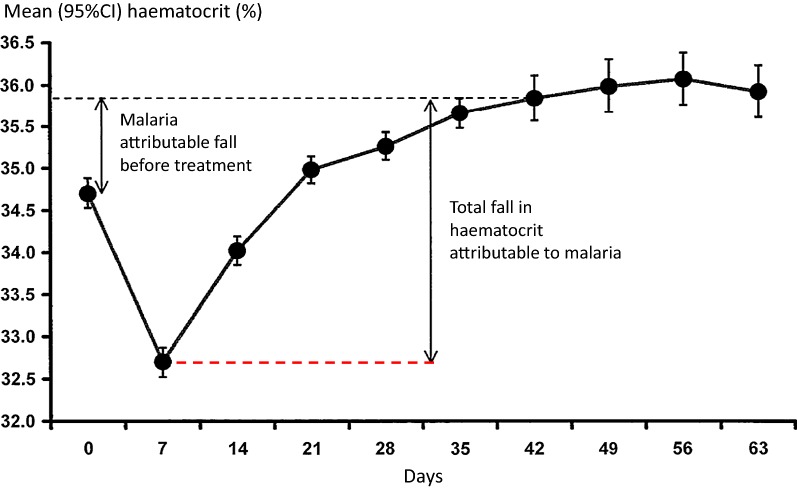
Development of anaemia and subsequent recovery in Karen patients with acute falciparum malaria treated on the Thailand-Myanmar border [33]

Anaemia develops rapidly in acute malaria so the majority of symptomatic patients have already lost at least 1 g of haemoglobin per decilitre (100 mL) of blood before presenting to medical attention. The liver and spleen enlarge rapidly. The anaemia is haemolytic so red cell indices are usually normal, haptoglobin and haemopexin concentrations are reduced, and unconjugated bilirubin may be raised. The leukocyte count is usually in the low-normal range and the platelet count is nearly always reduced. Slight elevations in transaminases may occur—rises in aspartate aminotransferase (AST, SGOT) result both from haemolysis and liver injury whereas alanine aminotransferase (ALT, SGPT) rises reflect liver injury only. With modern treatments (artemisinin-based combination treatments) defervescence occurs rapidly—and most patients have cleared fever and parasitaemia (assessed by microscopy) within 2 days [89]. In higher transmission settings, except in hyperparasitaemic children, the haemoglobin often starts to rise immediately, whereas in lower transmission settings, where patients have little or no immunity, the dyserythropoietic bone marrow is slow to recover, and in most patients the haemoglobin continues to fall reaching a nadir around 7 days after presentation [33] (Fig. 8). Thereafter anaemia gradually resolves and the haemoglobin concentration has returned to steady state values 4–6 weeks after the illness. The reduction in haemoglobin is approximately proportional to disease severity and duration of illness before treatment. Treatment with artemisinin derivatives attenuates the reduction in haemoglobin by providing rapid resolution of the disease and also by returning pitted red cells to the circulation. In a randomised comparison conducted in hyperparasitaemic children in Kinshasa, DRC, the initial fall in haemoglobin concentration following artesunate was less (a difference of 0.4 g/dL) than that following quinine [70] (Fig. 7). In contrast drug resistance is associated with increased anaemia both because of slow initial therapeutic responses and an increased risk of subsequent recrudescence. Both drug resistance and anaemia are independent risk factors for gametocytaemia in *P. falciparum* infections [96, 97], so in a context of worsening anti-malarial drug resistance it is common to see patients (often children) who present with anaemia and patent gametocytaemia.

**Fig. 9:**
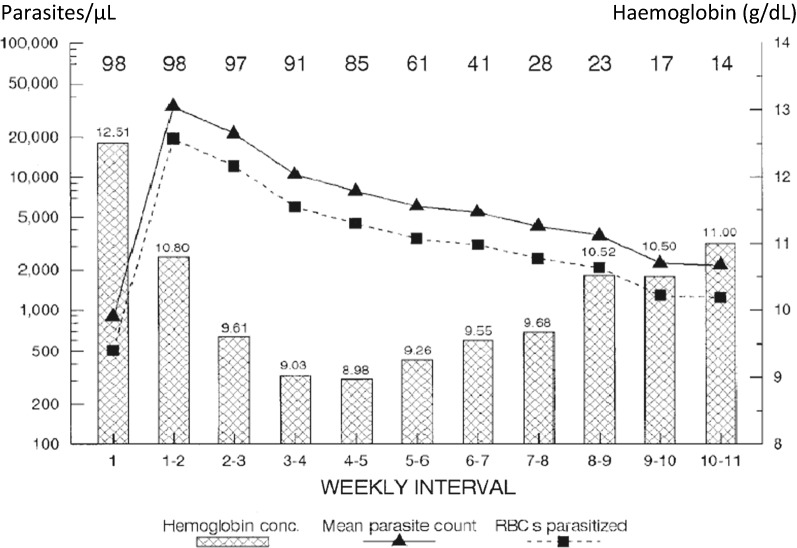
Untreated *Plasmodium vivax* infections in Australian army volunteers [102]. Numbers of subjects are given along the top. The haemoglobin concentrations reached a nadir during the 5th week of illness but then rose despite continuing parasitaemia

Anaemia may be regarded a clock of the infection, in that a patient presenting with acute malaria and a normal haematocrit (provided they are not dehydrated) cannot have been ill for many days. The cumulative impact of repeated malaria episodes was documented in detail during the malaria therapy era, which began nearly a century ago, when malaria was used to treat neurosyphilis and also in detailed volunteer studies conducted by the military [98–102]. In volunteers given malaria whose infections were untreated, haemoglobin concentrations declined rapidly initially over 1 to 2 weeks, and then declined more slowly with symptom resolution. Then after 4 to 5 weeks the haemoglobin concentration rose slowly despite persistent parasitaemia [102] (Fig. 9). These extensive and detailed observations demonstrated the important role of illness (manifest by fever) in causing anaemia. They showed how, with continued infection, tolerance to the infecting malaria parasites was induced, so that the inflammatory response subsided, and eventually effective erythropoiesis outstripped haemolysis.

### Severe malaria

Severe malaria is usually caused by *P. falciparum*, although *P. knowlesi* and occasionally *P. vivax* may also cause severe disease [93]. Severe malaria is a multi-system disease. Cerebral malaria (a diffuse symmetrical encephalopathy causing coma) is specific for *P. falciparum* infection, but kidney injury, metabolic acidosis, and severe anaemia may occur in all the malarias [93]. The pattern of vital organ dysfunction depends on age, pregnancy status, and, to a lesser extent, the level of transmission intensity [93]. Anaemia develops very rapidly in severe malaria. The initial most rapid decline in haematocrit observed in hospitalized patients reflects rehydration in those who are dehydrated and haemoconcentrated [103]. This has a time course of hours. It is followed by progressive haemolysis over the next few days without a significant erythropoietic response. The haemoglobin concentration commonly reaches a nadir around 1 week after admission [93]. In young children living in higher transmission settings the recovery is more rapid. In severe multisystem disease, the patient may lose 2 or more grams of haemoglobin per decilitre in the first 24 h of treatment. The dyserythropoietic bone marrow does not mount an effective reticulocyte response for several days.

The consequences of severe anaemia are an appropriate increase in cardiac index to maintain oxygen delivery. Very severe anaemia ultimately leads to tissue ischaemia and hypoxia, with a rise in lactate production and an increase in the lactate-pyruvate ratio [104–106]. Tissue ischaemia is also caused directly by severe falciparum malaria; the lactate-pyruvate ratio is also increased, and hyperlactataemia correlates strongly with outcome (vide infra), but the pathogenesis is different [93]. In patients with severe falciparum malaria and a high parasite burden the reduction in tissue oxygen delivery results from microvascular obstruction caused by sequestration, compounded by reduced red cell deformability and inter-erythrocytic adhesive forces [107, 108]. Thus, there are two overlapping causes of tissue hypoxia and hyperlactataemia which both occur in malaria:

- Severe anaemia which may result either from repeated uncomplicated infections with any of the malaria parasites, or a more fulminant haemolytic anaemia which is usually associated with severe falciparum malaria or sometimes blackwater fever.
- Reduced microvascular perfusion resulting from cytoadherence and inter-erythrocytic adhesion.

The first of these responds rapidly to blood transfusion.

### Anaemia and outcome in severe malaria

**Fig. 10:**
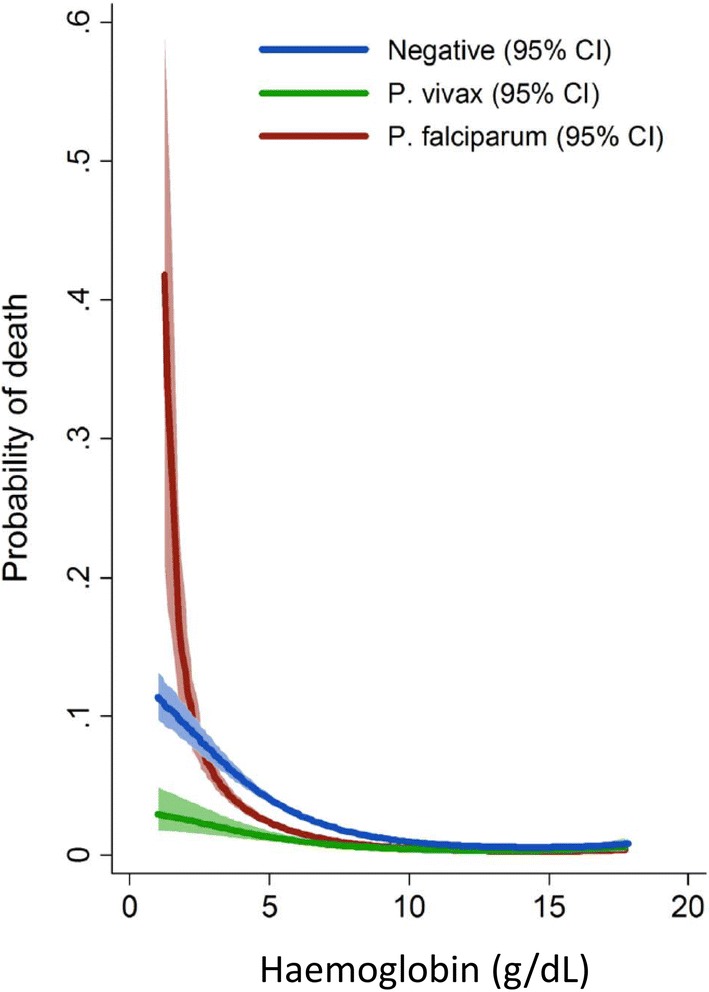
The relationship between admission haemoglobin and mortality in outpatients and hospitalized patients in Papua showing the substantially higher mortality of falciparum malaria with very severe anaemia compared with malaria caused by other Plasmodium species. Figures generated by multiple fractional polynomial regression analyses with the following covariables: Plasmodium species, hemoglobin, age, sex, ethnic group, and year. Bands represent 95% CIs (From Douglas et al. [9] with permission)

**Fig. 11:**
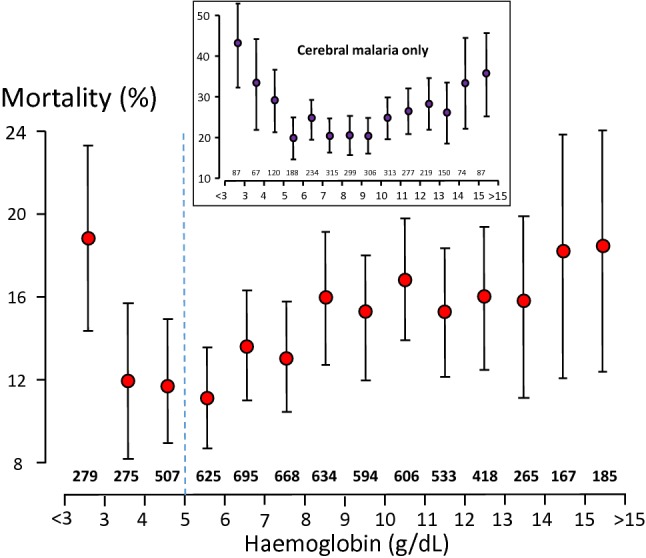
Overall relationship between haemoglobin concentrations on admission and outcomes in 6451 adults and children with severe falciparum malaria studied in Africa and Asia between 1980 and 2015 who were admitted with a parasite density of > 10,000/μL. Below 5 g/dL (vertical blue dashed line) anaemia itself was a criterion for severe malaria. Inset is shown this same relationship in the subgroup of 2729 patients with cerebral malaria and the same parasitaemia range

The relationship between haemoglobin concentration at presentation to hospital and outcome in falciparum malaria suggests that mortality rises sharply at levels below 3 g/dL (independent of other severe manifestations) [109, 110] (Figs. 10, 11). Severe anaemia is a prominent feature of all severe malaria but in areas of high transmission, where severe disease is confined to the first few years of life, it is the predominant manifestation [3, 11]. Consensus definitions of severe malaria have been agreed upon-which for the anaemia criterion is a haemoglobin below 5 g/dL in a patient with at least 10,000 parasites/μL [93]. Patients who fulfil the anaemia criterion for severe falciparum malaria, but have no other manifestations of severe malaria (i.e. cerebral, renal, metabolic or pulmonary dysfunction) have a much better prognosis than patients with one or more of these other manifestations [93]). Although most patients presenting with a parasite density of > 10,000 parasites/µL have malaria as the cause of their illness, this parasitaemia threshold for the “severe malaria” definition still means that some anaemic children with incidental parasitaemia and another infectious disease (usually sepsis) may be diagnosed as having severe malaria. Thus the severe anaemia criterion encompasses a broad range of presentations ranging from a fulminant disease with severe haemolytic anaemia to a sub-acute illness, often from recurrent or inadequately treated malaria, in which there has been progressive anaemia. The prognosis of the sub-acute presentation is much better as there has been time for physiological adaptation to anaemia (right shift of the oxygen dissociation curve) and there is a low risk of other vital organ dysfunction [93, 109]. In addition, the sequestered parasite biomass in these sub-acute presentations is much lower than in patients presenting with acute multi-organ dysfunction [110, 111]. Thus the mortality associated with severe malarial anaemia depends on many factors which include the degree of anaemia, age, transmission intensity, referral practices, access to and quality of health services, availability of safe cross-matched blood and delays in transfusion.

**Fig. 12:**
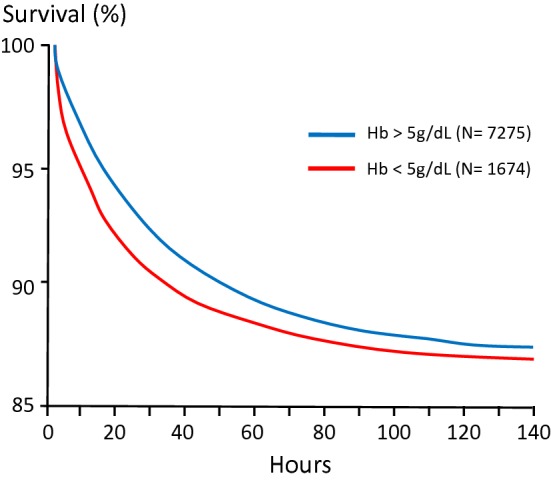
Time to death in children and adults admitted to hospitals in Asia and Africa with severe falciparum malaria

In published series of children admitted to hospital with severe malaria anaemia (including those with other severity manifestations) mortalities range from 2.6 to 10.3%—while mortalities as low as 0.5% have been reported in children with severe anaemia but without any other severity manifestation [106]. Those patients who are admitted with severe anaemia and do not survive succumb quickly, nearly half the deaths are within 12 h of admission (Fig. 12).

Above a haemoglobin concentration of 5 g/dL other manifestations are required for a diagnosis of “severe falciparum malaria”. In a very large series of over 8000 adults and children with strictly defined severe falciparum malaria studied in Asia and Africa over the past 37 years mortalities rose with increasing admission haemoglobin concentrations above 5 g/dL [112–117] (Fig. 11). A similar pattern was observed in the subgroup of patients admitted with cerebral malaria (Fig. 11). There are many potential confounders which may explain this finding, but a causal association cannot be excluded. Interestingly in the large FEAST fluid bolus trial [118], which enrolled children with both severe malaria and sepsis, the 8-h mortality of patients with mild anaemia (defined in the trial as haemoglobin 7–9.9 g/dL) was higher among patients who still received a blood transfusion (8/35, 23%) than it was in the patients in the same category who were not transfused (29/808, 4%); a risk ratio of 6.4 (95% CI 3.1–12.9), P < 0.0001 [118]. This large difference was ascribed, very reasonably, to likely severity indicators which prompted a transfusion in mildly anaemic children, in whom it is generally not warranted. But it does raise the possibility of a causal association in patients with severe malaria. If this were confirmed it would mean that mild anaemia reduces the probability of a fatal outcome in patients with severe falciparum malaria (and vital organ dysfunction). It does not mean that anaemia could be beneficial overall—indeed anaemia is clearly harmful—but that within the subgroup of patients with falciparum malaria who have developed vital organ dysfunction it is possible that anaemia protects against death. If a causal association were confirmed then how could it be explained?

The ability of the circulatory system to transport oxygen to tissues and organs is determined by the cardiac index, the rheological properties of blood and the architecture of the microvasculature. Cardiac index is increased in anaemia. Blood, a suspension of cells in proteinaceous fluid, has complex rheological properties with a non-linear relationship between pressure and flow (shear stress and shear rate respectively i.e. non-Newtonian fluid mechanics) which could be altered in severe malaria. Increasing haematocrit is associated with a linear increase in oxygen carriage but a non-linear increase in apparent viscosity. As a consequence there is an optimum haematocrit for oxygen delivery [119]. The dependence of blood viscosity on haematocrit is greatest at the low shear rates encountered in the venous circulation, where sequestration of *P. falciparum* infected erythrocytes begins [120, 121]. Finally, the architecture of the microcirculation is markedly disrupted by sequestration. Cytoadherence, the fundamental pathological process in severe malaria which causes sequestration has been shown to be reduced by haemodilution. In an ex vivo system there was a 5- and 12-fold increase in *P. falciparum* infected erythrocyte rolling and adhesion, respectively, when haematocrit increased from 10 to 30%, as a result of changes in shear rate [122]. The optimum haematocrit is not known in severe malaria, but red cell adherence to vascular endothelium, and to other erythrocytes is likely to lower it, and reduced erythrocyte deformability may reduce it further [121, 123]. Thus reducing the density of erythrocytes (i.e. anaemia) might improve microvascular perfusion in patients with severe malaria. If so that would increase oxygen delivery, until it was outweighed by the reduction in oxygen carriage.

Alternatively there is no causal relationship. Severe anaemia may simply reflect duration of illness, and thus control of a severe infection or series of infections without intervening lethal vital organ dysfunction. The observed relationship between admission haematocrit and outcome of severe malaria might be explained entirely by other covariate relationships. Clearly this is an important question requiring further study to inform treatment guidelines and patient management.

### Blackwater fever

This condition is well described but still poorly understood [93]. Blackwater fever means sudden massive haemolysis with fever and haemoglobinuria. The urine is black and, if the haemolysis is extensive, the patient has a pale, slate-grey appearance. Blackwater fever may be part of severe malaria [124–126]. Death may occur from severe anaemia or from acute renal failure. Haemoglobinuria may also occur in otherwise uncomplicated infections. Blackwater fever has historically been linked to quinine use, and it may occur in glucose-6-phosphate dehydrogenase deficiency with febrile illnesses such as malaria or following ingestion of oxidants (notably radical cure primaquine regimens) [127].

### Post-artesunate haemolytic anaemia

There are several reports, mainly from temperate countries describing returned travellers, of late haemolytic anaemia occurring 1–3 weeks after parenteral artesunate treatment of hyperparasitaemic falciparum malaria. Most cases followed intravenous artesunate, although some were reported after intramuscular artemether, intrarectal artesunate and oral artemisinin derivatives. No deaths have been reported, but blood transfusion was often required. A recent review of published data, from non-immune and semi-immune patients, estimated the incidence of late haemolysis after intravenous artesunate to be 13% (95% CI 9–18%) and the requirement for a blood transfusion at 9% (95% CI 6–14%). Most of the data are from case reports and case definitions have varied substantially [67–69, 128]. However, in African children post-artesunate haemolytic anaemia occurs in less than 1% of cases [70] (Fig. 7). Post-artesunate haemolytic anaemia has been attributed to the pitting of drug-damaged malaria parasites from infected erythrocytes [67]. These once-infected red blood cells (*oi*-RBC) have a much shorter survival time (7–14 days) compared with normal erythrocytes in healthy subjects (120 days) or in patients following severe malaria (44 days) [66, 128]. In the French series, a threshold of 180 once-infected erythrocytes/µL discriminated patients with delayed haemolysis with 89% sensitivity and 83% specificity [67]. The shorter survival of *oi*-RBC following artesunate probably reflects drug killing and then pitting of developed ring form parasites whereas background (and quinine associated) pitting may only occur for very young ring stages shortly after merozoite invasion with correspondingly less damage to the erythrocytes. The rapid and synchronous elimination of these *oi*-RBC from the circulation 1 to 2 weeks after the start of anti-malarial treatment results in haemolytic anaemia, which in some cases can be marked [67].

## Management

### Severe malaria

Severe anaemia (haemoglobin < 5 g/dL) requires blood transfusion which can be life-saving [129, 130]. The lower the haemoglobin the greater the need for transfusion. If there are other features of severe malaria such as acidotic breathing (respiratory distress) or coma, together with severe anemia then transfusion is more urgent. Respiratory distress with severe anaemia is often a sign of impending death [93, 131]. The rate of transfusion is titrated according to vital signs. In children, the WHO recommends 20 mls/kg of whole blood to be given over 4 h, or 10mls/kg of packed cells (although this is often unavailable). In low transmission settings malaria treatment guidelines recommend transfusion in severe malaria if the haemoglobin is < 7 g/dL (haematocrit 20%), whereas in higher transmission settings the recommended threshold is 5 g/dL (haematocrit 15%) [93]. The haemoglobin transfusion thresholds for acute malaria differ slightly from other WHO guidelines for the clinical use of blood. These recommend transfusion if the haemoglobin concentration is 4 g/dL or less (or haematocrit 12%), whatever the clinical condition of the patient, but they also recommend transfusion for haemoglobin concentrations of 4–6 g/dL (or haematocrits of 13–18%) if clinical features of any the following are present: hypoxia, acidosis, impaired consciousness or hyperparasitaemia (> 20%) [132, 133]. None of these transfusion recommendations are based on solid evidence [134]. Transfusion thresholds in malaria were set intentionally slightly higher than for some other conditions [135] because haemoglobin concentrations usually fall rapidly in severe malaria. Thus, to avoid falling into the danger zone it has been thought better to order blood for transfusion sooner rather than later. But what is the danger zone? And are these pragmatic thresholds correct? Retrospective observations have the disadvantage that many factors determine whether or not a patient with severe malaria receives a timely blood transfusion, and several of these factors could affect outcomes independently, so prospective randomized trials are under way to try and answer these questions [134].

The anti-malarial treatment of choice for severe malaria is parenteral artesunate [89, 93, 114, 116]. When the patient can swallow reliably this should be followed by a full course of an oral artemisinin-based combination therapy. Anti-malarial treatment should not be delayed by transfusion.

In areas where the HIV prevalence is high severe anaemia with malaria is more likely in HIV positive patients [136], and in high malaria transmission settings concomitant HIV exacerbates the anaemia that occurs in infancy after 3 months of age [137]. In these high transmission settings children admitted to hospital with severe anaemia have an increased risk of readmission with severe anaemia, and they also have an increased risk of dying in the following months [10]. Prevention of malaria reinfection with slowly eliminated anti-malarial drugs reduces these risks [138].

### Blackwater fever

Blackwater fever results from massive haemolysis sufficient to cause haemoglobinuria. The management of blackwater fever anaemia is with blood transfusion [93]. Anti-malarial treatment should not be withheld. Steroids are ineffective. Some cases result from oxidant haemolysis in G6PD deficiency. In these cases the precipitant should be withdrawn and adequate hydration ensured. Blackwater fever patients, who are not G6PD deficient, are notoriously difficult to cross match. Acute kidney injury is an important complication.

### Anaemia in uncomplicated malaria

A promptly treated discrete episode of malaria in a patient with a pre-morbid normal haemoglobin is unlikely to result in clinically significant anaemia. It is the cumulative impact of repeated illness from recurrent malaria that is the main cause. In areas where *P. vivax* is endemic frequently recurring illness caused by repeated relapse is the main contributor to malarial anaemia in childhood [8, 9]. In areas where *P. falciparum* is endemic frequent infections or repeated treatment failure cause anaemia. The prevalence of anaemia therefore increases with transmission intensity, and where anti-malarial drug resistance compromises drug efficacy [139]. In areas of high transmission the greatest burden is in infants [3]. Prompt effective artemisinin combination treatment is currently the cornerstone of management of the individual acute illness [89], but prevention of repeated or recurrent infection is the key to reducing malarial anaemia at the community level.

### Malaria in pregnancy

Anaemia is common in pregnancy in tropical regions. Malaria is a major contributor to anaemia in pregnancy [13, 14, 139]. The risks of anaemia increase as the pregnancy progresses although severe haemolytic anaemia may occur during the middle trimester in high transmission settings. Concomitant HIV infection exacerbates malarial anaemia in pregnancy. High maternal parasitaemias are associated with fetal and newborn anaemia [6]. Even asymptomatic infection in the mother is harmful for the fetus and reduces birthweight. Symptomatic infections need prompt treatment, but prevention is better for both mother and baby. This can be achieved by chemoprophylaxis, although there is currently no satisfactory safe drug for falciparum malaria prevention. Chloroquine is regarded as safe in pregnancy and is still effective in preventing the non-falciparum malarias in most areas. The more widely used approach in higher transmission settings is intermittent preventive treatment which involves giving treatment doses at approximately 1 month intervals starting from 13 weeks gestation, or initial presentation at the antenatal clinic—whichever is later [89]. Sulfadoxine–pyrimethamine is the most widely used drug although this is increasingly challenged by resistance. The WHO recommends that in malaria-endemic areas of Africa, intermittent preventive treatment with SP should be provided to all pregnant women (SP-IPTp) as part of antenatal care, unless they are HIV coinfected and already receiving co-trimoxazole prophylaxis. Dosing should start in the second trimester and doses should be given at least 1 month apart, with the objective of ensuring that at least three doses are received [89]. Pregnant women in malaria endemic areas should also receive iron and folate supplementation according to standard guidelines.
